# Effect of Carboxyl Group Position on Assembly Behavior and Structure of Hydrocarbon Oil–Carboxylic Acid Compound Collector on Low-Rank Coal Surface: Sum-Frequency Vibration Spectroscopy and Coarse-Grained Molecular Dynamics Simulation Study

**DOI:** 10.3390/molecules29051034

**Published:** 2024-02-28

**Authors:** Zechen Liu, Xianshu Dong, Yinfei Liao, Yuping Fan, Yijun Cao

**Affiliations:** 1 School of Mining Engineering, Taiyuan University of Technology, Taiyuan 030024, China; fanyuping@tyut.edu.cn; 2 Chinese National Engineering Research Center of Coal Preparation and Purification, China University of Mining and Technology, Xuzhou 221116, China; ruiyin@126.com (Y.L.); yijuncao@126.com (Y.C.); 3 Zhongyuan Critical Metals Laboratory, Zhengzhou University, Zhengzhou 450001, China

**Keywords:** compound collector, sum-frequency vibration spectroscopy, coarse-grained molecular dynamics simulation, interface assembly, carboxyl group position

## Abstract

In this work, the assembly behavior and structure of a compound collector with different carboxyl group positions at the low-rank coal (LRC)–water interface were investigated through coarse-grained molecular dynamics simulation (CGMD) combined with sum-frequency vibration spectroscopy (SFG). The choice of compound collector was dodecane +decanoic acid (D-DA) and dodecane +2-butyl octanoic acid (D-BA). CGMD results showed that the carboxyl group at the carbon chain’s middle can better control the assembly process between carboxylic acid and D molecules. SFG research found that the carboxyl group at the carbon chain’s termination had a greater impact on the displacement of the methyl/methylene symmetric stretching vibration peak, while the carboxyl group at the carbon chain’s middle had a greater impact on the displacement of the methyl/methylene asymmetric stretching vibration peak. The spatial angle calculation results revealed that the methyl group’s orientation angle in the D-BA molecule was smaller and the carboxyl group’s orientation angle in the BA molecule was bigger, indicating that D-BA spread more flatly on the LRC surface than D-DA. This meant that the assembled structure had a larger effective adsorption area on the LRC surface. The flotation studies also verified that the assembly behavior and structure of D-BA with the carboxyl group at the carbon chain’s middle at the LRC–water interface were more conducive to the improvement of flotation efficiency. The study of interface assembly behavior and structure by CGMD combined with SFG is crucial for the creation of effective compound collectors.

## 1. Introduction

A compound with a carboxylic group (–COOH) in the molecule is called a carboxylic acid. The carboxylic carbon atom makes three σ bonds in sp2 hybrid orbitals, two of which are with two oxygen atoms and one with the hydrocarbon group. The remaining p electron joins the oxygen atom in the carboxylic group to form the π bond of C=O, but the oxygen in the –OH portion of the carboxylic group contains a pair of unshared electrons that can join the π bond to form a p-π conjugated system. The oxygen atom in the –OH group advances its electron cloud towards the carbonyl group due to p-π conjugation, as well as the oxygen atom being nearer to the electron cloud between O–H. This strengthens the polarity of the O–H bond and makes it easier for the H atom to dissociate [1]. Based on the properties of the carboxyl group, many scholars in the field of flotation use chemicals containing the carboxyl group as collector components to efficiently separate useful minerals. Cao et al. [2] used a mixture of oleic, linoleic, and linolenic acids as collectors to increase the flotation recovery of phosphate. Keith Quast [3] demonstrated that the 18-carbon unsaturated fatty acid mixtures were an ideal flotation agent for recovering oxidized minerals with high mineral recovery rates. Similarly, for the flotation of low-rank coal (LRC), carboxylic acid agents are added based on traditional oil collectors to form strong hydrogen bonds between carboxylic groups and the LRC surface, so as to achieve the efficient separation of LRC. Tian et al. [4] discovered that the flotation effect of LRC in the presence of carboxylic acid was superior to that of alkane. Liu et al. [5,6] systematically studied the active mechanism of the mass ratio and addition sequence of the carboxylic acid composite collector on LRC flotation. For the choice of carboxylic acid, most carboxylic acid reagents were discovered to disperse their carboxylic group near the end of the carbon chain, and the reason for this was to make the carbon chain better in the collection performance. However, when the carbon chain’s carboxyl group is in the middle, what about the harvesting effect of low-rank coal? In other words, the structure–activity relationship of the carboxyl group position in LRC flotation is worthy of further study, which has significant ramifications for the development and use of compound collectors for LRC.

In the field of interface chemistry, experts and scholars have proposed the interface assembly mechanism of mixed surfactants in solution. In the mineral processing of metal ores, studies have shown that composite collectors have an assembly process on mineral surfaces [7,8,9]. In recent years, Han et al. [10,11] proposed the assembly mechanism of different types of collector molecules at mineral surface interfaces, including the concepts of the assembly of different electrical collectors and interface hydrophobic assembly. Cui et al. [12,13] also used density functional theory to study the arrangement structure and arrangement mode of mineral collector molecules with different structures at the solid–liquid interface. However, there are few studies on the interfacial assembly mechanism of LRC compounding collector molecules, which are not deep enough. Regarding how the compound collector and LRC interact, according to the prevalent theory now in use, the LRC surface is positively impacted by the synergistic effects of the different medicinal compounds in the compound collector, and the co-adsorption structure that results encourages the adsorption of the entire reagent [14,15]. When the collector molecules in the solid–liquid interface system are adsorbing to the LRC surface, the different structures of the chemical molecules themselves will lead to different assembly structures, which is related to the interface synergy effect, and ultimately affects the LRC flotation efficiency. Therefore, the study of the interface assembly structure and behavior of chemical molecules is the key to realizing the controllable adsorption and efficient development of compound collectors on the LRC surface.

A breakthrough has been made in the simulation and characterization of interface assembly, but most of the research focuses on polymer material structure, nanomaterial structure, biofilm structure, and mineral crystal surface. However, the interfacial assembly mechanism of LRC collector molecules has not been studied, and the assembly structure and behavior involved have not been deeply explored. It is encouraging that the research on the interface assembly structure and system has become more and more mature in other fields, and its simulation and detection methods are expected to bring a reference for the research on the surface assembly structure and mechanism of LRC. Coarse-grained molecular dynamics simulation (CGMD) has been applied to the study of self-assembly systems [16,17,18], enabling the study of assembly processes at larger time scales and spatial scales. Additionally, Liu et al. [19] used CGMD to investigate the spreading characteristics of collector droplets at the coal–water interface. Due to the macroscopic process of molecular assembly, besides simulation and prediction, it is characterized by various methods, such as scanning probe microscopy, electrochemistry, spectroscopy, X-ray diffraction, and transmission electron microscopy. The arrangement, orientation, and spatial conformation of the molecules on the solid support can be analyzed by the sum frequency generation spectrometer (SFG). Champika Weeraman et al. [20] used SFG technology to demonstrate the relationship between the molecular conformation of dodecyl mercaptan ligands that have been chemically adsorbed and the surface curvature of spherical gold nanoparticles. Wang et al. [21] used SFG technology to study the adsorption state of dodecylamine on the quartz surface at various PH levels. All these provided the possibility for studying the compound collector’s structure and assembly behavior on the LRC surface.

Therefore, this paper mainly studied the assembly behavior and structure of the compound collector made up of hydrocarbon oil and carboxylic acid at the LRC–water interface through SFG combined with CGMD, clarified the mechanism of carboxylic acid at different carboxylic group positions for the assembly mechanism of the compound collector, and provided a theoretical reference and guidance for the creation of a compound collector and the regulation of molecule assembly on the LRC surface.

## 2. Results and Discussion

### 2.1. CGMD Simulation of Assembly Behavior of Carboxylic Acid Compound Collector with Different Carboxylic Group Positions at Low-Rank Coal/Water Interface

#### 2.1.1. Assembly Process Analysis

**Figure 1:**
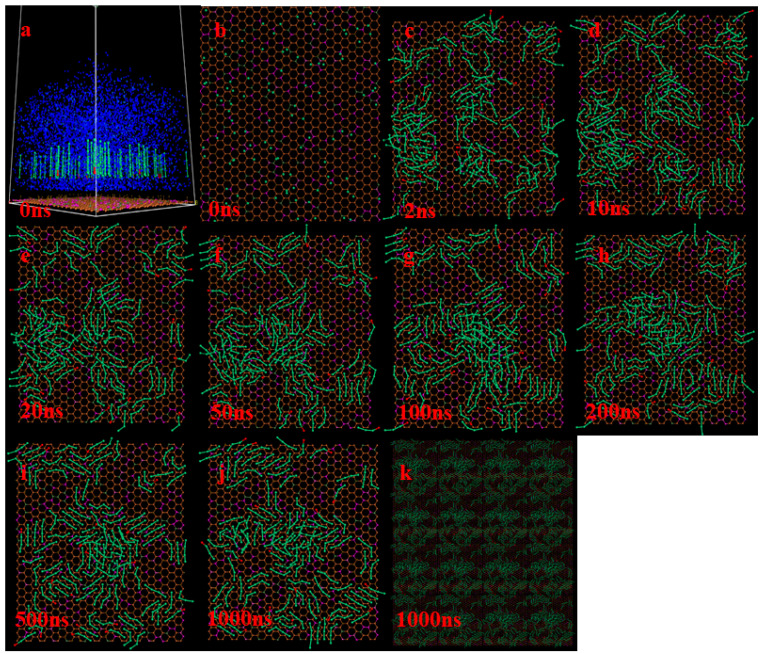
Adsorption and assembly structure of the compound collector molecule D-DA at the coal–water interface. Before 50 ns, D-DA molecules gradually diffuse and rearrange in the form of individuals or clusters after adhering to the LRC surface (**b**–**f**). 50ns-1000ns, D-DA molecules complete the rearrangement assembly (**f**–**j**). The blue is the coarse water particles, long chain alkanes are represented by a green bat model, the carboxyl group is represented by a red ball. In (**a**), the boundaries of cells are represented by white lines. (**a**) is the overall view of the simulation system, and (**b**) is the top view of (**a**). (**k**) is a quadruple graph of (**j**) in the X × Y boundary range. Note that (**b**–**k**), water is not shown to better show the assembly of D-DA.

**Figure 2:**
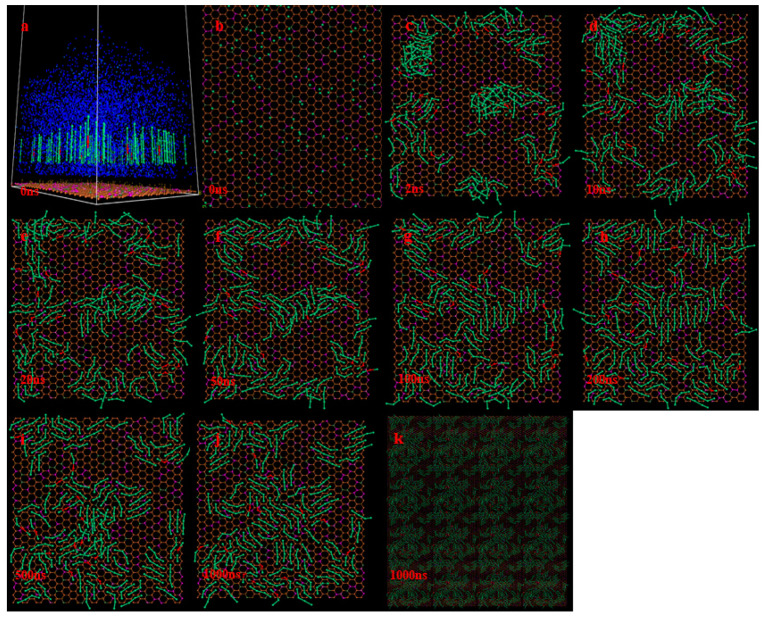
Adsorption and assembly structure of the compound collector molecule D-BA at the coal–water interface. Before 50 ns, D-BA molecules gradually diffuse and rearrange in the form of individuals or clusters after adhering to the LRC surface (**b**–**f**). 50ns-1000ns, D-BA molecules complete the re-arrangement assembly, and the adsorption of molecules in the first layer is more orderly (**f**–**j**). The blue is the coarse water particles, long chain alkanes are represented by a green bat model, the carboxyl group is represented by a red ball. In (**a**), the boundaries of cells are represented by white lines. (**a**) is the overall view of the simulation system, and (**b**) is the top view of (**a**). (**k**) is a quadruple graph of (**j**) in the X × Y boundary range. Note that (**b**–**k**), water is not shown to better show the assembly of D-BA.

The trajectory results of CGMD simulation calculation were analyzed, and the coal–water interface adsorption structure diagrams at different time nodes from 0 ns to 1000 ns were selected to determine the accumulation, adsorption behavior, and assembly process of compound collector molecules at different carboxy-group positions at the LRC–water interface. Figure 1 and Figure 2 respectively show the adsorption structures of the compound collector molecules with different carboxyl group positions at different time nodes of the coal–water interface, where because of the three-dimensional periodic boundary conditions, for ease of observation, Figure 1k or Figure 2k is a quadruple graph of Figure 1j or Figure 2j in the X × Y boundary range. By comparing the molecular structure of the reagent at different time nodes, it is found that in the process of adsorption to the LRC surface, the compound collector molecules are forced between molecules to form an assembly process (Figure 1c–j or Figure 2c–j), and the assembly structure tends to form semi-ordered clusters of several or a dozen molecules to seek to minimize the dynamic energy. Before 50 ns, that is, when the compound collector molecules gradually diffuse and rearrange in the form of individuals or clusters after adhering to the LRC surface, the LRC surface has a larger spreading area for the D-DA with a carboxyl group at the carbon chain’s termination than the D-BA with a carboxyl group at the carbon chain’s middle. It shows that the molecular rearrangement process of D-DA on the LRC surface occurs more quickly. When the final assembly structure is formed, compared with Figure 1j or Figure 2j of the two figures, the adsorption of the molecules in the D-BA is more orderly in the first layer. Compared with D-DA, there are fewer crossed molecules on its surface. The assembly process between carboxylic acid molecules and D molecules seems to be better controlled when the carboxylic group is in the carbon chain’s middle. Because the D molecule and carboxyl group have a stronger non-bonding connection, the methyl group at the other end of the carboxylic acid molecule is arranged far away from the D molecule, making the assembly structure less parallel and orderly than that of D-BA.

#### 2.1.2. Analysis of Action Mechanism during Assembly

**Figure 3:**
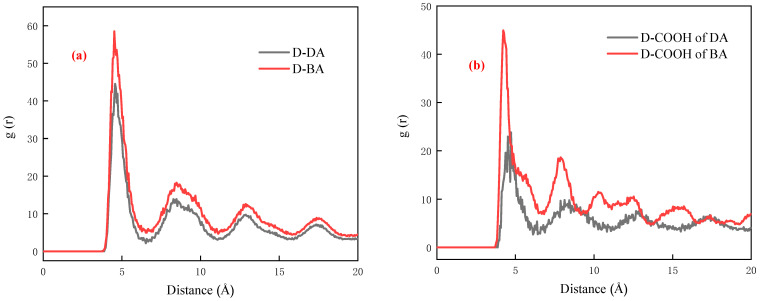
(**a**) The RDF curves between the D and carboxylic acid molecules in different kinds of compound collectors. (**b**) The RDF curves between the D and carboxyl groups in different kinds of compound collectors.

The last 200 ns trajectory file was selected to analyze the interaction energy and radial distribution function (RDF) [22] between the D and carboxylic acid molecules in different kinds of compound collectors, and explore the action mechanism of different kinds of compound collectors in the process of coal–water interface assembly. As seen by the RDF outcomes in Figure 3a, for the compound collector with different carboxyl group locations, all RDF curves have two significant peaks at a distance of 10.0 Å, the first peak near 5.0 Å and the second peak near 8.5 Å. If the distance between two molecules from the center to the center is less than 10 Å, they are considered neighbors and can form small clusters or aggregates [23], so the two significant peaks in the RDF curve at a distance of 10.0 Å represent the possibility of carboxylic acid molecules forming clusters around the D molecule. For the two peaks of RDF, the order of peak intensity between carboxylic acid molecules and D molecules at different carboxylic group positions is as follows: DA < BA. Moreover, Figure 3b shows the RDF curve between carboxyl groups and D molecules. The RDF peak intensity between carboxyl groups and D molecules is also stronger, and the peak distance of RDF peaks between different carboxyl groups and D molecules is 8.125 Å (D-DA) and 7.725 Å (D-BA), respectively. It shows that the BA molecule with a middle-placed carboxyl group on its carbon chain interacts more strongly with the D molecule as a whole, and the interaction distance is also closer. Therefore, when forming the final assembly structure, the first layer adsorption of the molecules in the D-BA is more parallel and ordered.

In order to better explain the interaction in the compound collector between the D and the carboxylic acid molecules, the interaction energy between the D and carboxylic acid molecules was calculated. The formula for calculating the interaction energy ($E_{\mathrm{inter}\;\mathrm{dodecane}\;\&\;\mathrm{acid}}$) between the D molecule and the carboxylic acid molecule is:

(1) $$E_{\mathrm{inter}\;dodecane\;\&\;\mathrm{acid}\;}=\left(\begin{array}{l} E_{total}-E_{dodecane+coal-water}-E_{acid+coal-water}\\ -E_{dodecane}-E_{acid}+E_{coal-water}+E_{dodecane+acid} \end{array}\right)/2$$

$E_{total}$, $E_{dodecane}$, $E_{acid}$, and $E_{coal-water}$ are the energy of the entire system as well as the energy of dodecane molecules, carboxylic acid molecules, and the entire coal–water system. $E_{dodecane+coal-water}$, $E_{acid+coal-water}$, and $E_{dodecane+acid}$ are the total energy of dodecane + coal/water, carboxylic acid + coal/water, and dodecane + carboxylic acid, respectively. Dodecane and carboxylic acid molecules interact with each other more strongly the higher the absolute value of $E_{\mathrm{inter}\;\mathrm{dodecane}\;\&\;\mathrm{acid}}$. The interaction energies of $E_{\mathrm{inter}\;\mathrm{dodecane}\;\&\;\mathrm{DA}}$ and $E_{\mathrm{inter}\;\mathrm{dodecane}\;\&\;\mathrm{BA}}$ are −114.42 and −116.35 kcal/mol, respectively. At the coal–water interface, it can be seen that the interaction energies between carboxylic acid molecules at different carboxylic group positions and D molecules have little difference, while the absolute values of interaction energies between BA molecules and D molecules are larger than that between DA molecules and D molecules. The results show that carboxylic acid molecules with a carboxyl group in the middle of the carbon chain have a stronger interaction with the D molecule at the coal–water interface, which also complies with the observed assembly structure and RDF calculation results.

### 2.2. Assembly Structure of Carboxylic Acid Compound Collector with Different Carboxylic Group Positions at LRC/Water Interface

#### 2.2.1. SFG Spectral Analysis

**Figure 4:**
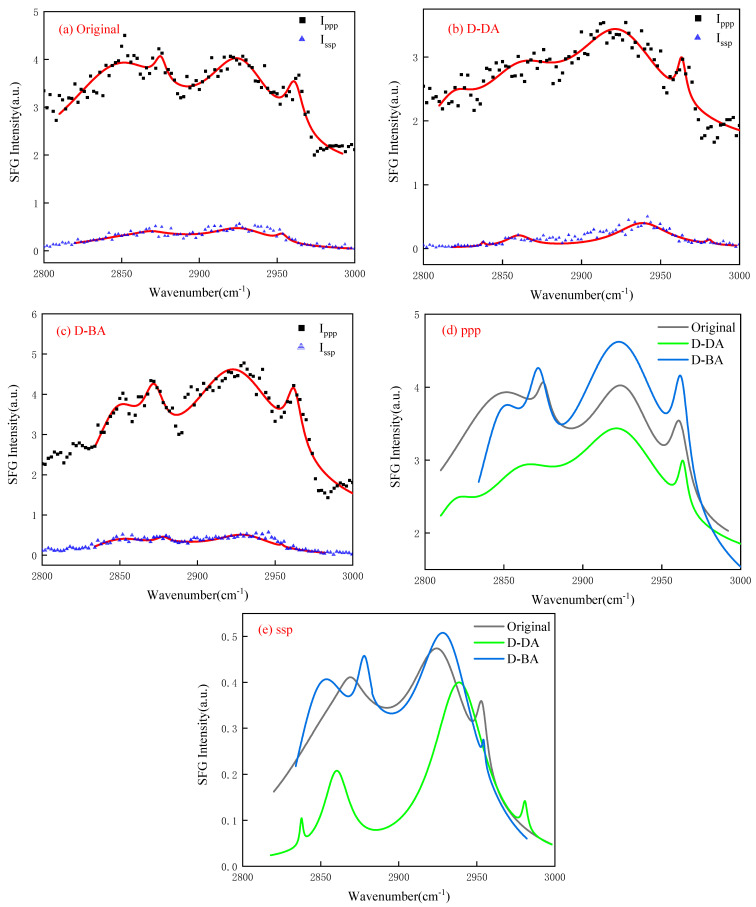
(**a**) Sum-frequency vibration spectra of different polarization states of coal chip without adsorbing any reagent; (**b**,**c**) Sum-frequency vibration spectra of compound collectors with different carboxyl positions adsorbed on the surface of coal chip in different polarization states; (**d**,**e**) The fitted total peak lines by the sum-frequency vibration spectra of different polarization states; red lines are the fitted total peak lines.

As depicted in Figure 4, the SFG spectra of the compound collector with different carboxyl group positions were obtained by fitting under different polarization states after adsorption on the surface of the coal chip. For contrast, Figure 4a displays the SFG spectra in various polarization conditions, showing no absorption of any substance by the coal chip. In the initial coal chip, when in a ppp polarization state, the C–H vibration exhibits distinct peak vibrations in these categories: the methyl group’s symmetric stretching vibration peak (CH_3, ss_, 2875 cm^−1^) and the asymmetric stretching vibration peak (CH_3, as_, 2961 cm^−1^); the methylene groups’ symmetric stretching vibration peaks (CH_2, ss_, 2852 cm^−1^) and the asymmetric stretching vibration peaks (CH_2, as_, 2924 cm^−1^). During ssp’s polarization state, the primary group vibration peaks for C–H are CH_3, ss_ (2869 cm^−1^), CH_3, as_ (2955 cm^−1^), and CH_2, as_ (2924 cm^−1^) [24,25,26]. Generally, in the ssp polarization state, the SFG signal from the solid chip surface is faint, with the methylene group exhibiting only a slight asymmetric stretching vibration. In contrast to the ssp polarization state, the ppp polarization state exhibits significantly greater spectral intensity. Additionally, the compound collector’s abundance of methylene groups, owing to their varied locations on the carbon chain, results in a broader peak width for the methylene group vibration peak compared to the methyl group [27].

Upon adsorbing the compound collector, which has varied positions of carboxyl groups, onto the solid chip, Figure 4d,e and Table 1 displays the respective vibration peaks of these groups. Combined with the chart, it can be seen that the main difference in the ppp polarization state is the detected CH_2, ss_ vibration peak positions, which are 2829 cm^−1^ (D-DA) and 2852 cm^−1^ (D-BA), respectively. Compared with the original chip, the CH_2, ss_ vibration peak positions on the chip surface after D-DA treatment are more shifted. This also shows that the DA molecule with a carboxyl group at the carbon chain’s termination requires a lower energy for the assembly process of the D molecule, and the BA molecule with a carboxyl group at the carbon chain’s middle requires a higher energy for the assembly process due to its strong non-bond interaction with D molecule. At the same time, in the polarization state of ppp and ssp, the wave number of the symmetric stretching vibration peak (ss) of the chip treated with D-DA is smaller than that of the chip treated with D-BA. The wave number of the asymmetric stretching vibration peak (as) of the chip treated with D-BA is smaller than that of the chip treated with D-DA, which is also caused by the difference in the position of the carboxyl group. The carboxyl group has a great influence on the vibration frequency of the methyl or methylene groups around it due to its strong electron absorption. Therefore, the position of the carboxyl group at the carbon chain’s termination has a greater influence on the displacement of methyl/methylene symmetric stretching vibration peak, and the position of the carboxyl group at the carbon chain’s middle has a greater influence on the displacement of the methyl/methylene asymmetric stretching vibration peak.

#### 2.2.2. Evaluation of Orientation Angle and Conformational Change in Carboxyl Groups at LRC/Water Interface

**Figure 5:**
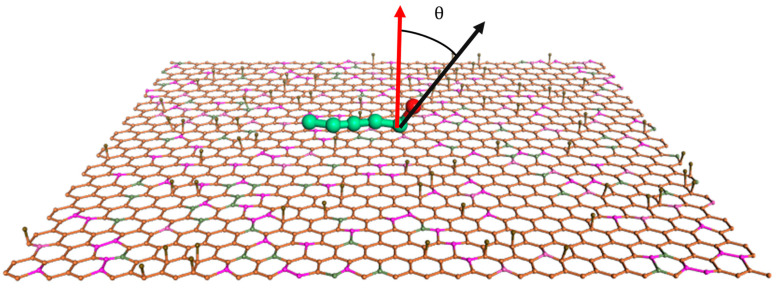
Schematic diagram of the azimuth angle of the carboxyl group (The red arrow indicates the normal vector, and the black arrow indicates the vector from the carboxyl group adjacent coarse particles to the carboxyl group coarse particles).

**Figure 6:**
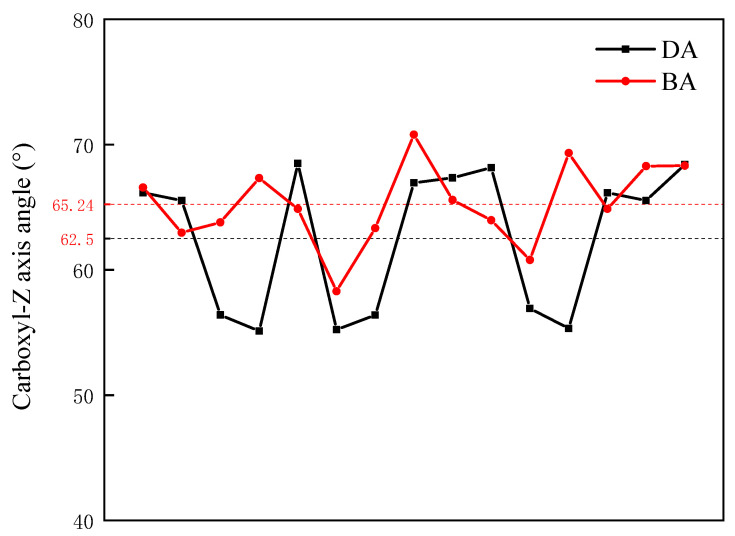
The orientation distribution of carboxyl groups of carboxylic acid molecules with different carboxyl positions.

To better comprehend how various types of compound collector molecules differ in their assembly structures at the coal–water interface, the final output dynamic calculation structure analysis of each system was selected. The orientation of polar groups in different compound collector molecules in space is different during assembly. Based on the characteristics of coarse-grained modeling, the orientation distributions of polar groups of different carboxylic acid molecules relative to the model plane of LRC were calculated. The directional angle θ is referred to as the angle formed by the normal vector and the vector from the carboxyl group adjacent coarse particles to the carboxyl group coarse particles (see Figure 5). Figure 6 shows the distribution of carboxyl groups of carboxylic acid molecules at different carboxyl group positions relative to the LRC plane. It can be seen that the carboxyl groups in carboxylic acid molecules at different carboxyl group positions are 62.5° (DA) and 65.24° (BA), respectively. The orientation distribution angle of carboxyl groups in BA molecules is larger, indicating that BA molecules with carboxyl groups at the carbon chain’s middle have a flatter spreading angle on the LRC surface than DA molecules with carboxyl groups at the carbon chain’s termination, and the carboxyl groups are more closely related to the surface of LRC, and the adsorption with low-rank coal surfaces is more stable in the polar water phase. This also indicates that the carboxyl group at the carbon chain’s middle interacts more strongly with the LRC surface.

#### 2.2.3. Evaluation of Orientation Angle and Conformational Change in Methyl Groups at LRC/Water Interface

In the above section, the overall orientation angle and conformation changes in carboxylic acid groups in carboxylic acid molecules were calculated by simulation. To better explain the overall interfacial adsorption conformation of compound collector molecules, the methyl group orientation angle distribution at the gas–solid interface after different kinds of compound collector adsorbed on the surface of solid chip was calculated by combining the SFG spectrogram characteristics and the formula for measuring the orientation angle of interfacial molecular groups. The orientation angles corresponding to the methyl groups on the surface of the solid chip are 43.06° (Original), 49.30° (D-DA), and 46.74° (D-BA), respectively, before and after the adsorption of the compound collector at different carboxyl group positions. Combined with the orientation angle distribution of the carboxyl groups obtained by CGMD, although the overall orientation angle of the carboxyl group of BA molecule is larger, that is, it is more parallel to the LRC surface because the carboxyl group is in the carbon chain’s middle, the carbon chain on both sides of the carboxyl group will form a certain angle, so there is a minor orientation angle for the methyl group on the chip surface after D-BA treatment. That is, the methyl groups of some BA molecules tend to be vertically distributed in the direction of the LRC surface. In general, D-BA spreads to be more flat on the LRC surface than D-DA, and the effective adsorption area of the assembled structure on the LRC surface is larger.

#### 2.2.4. AFM Adsorption Morphology Characterization

**Figure 7:**
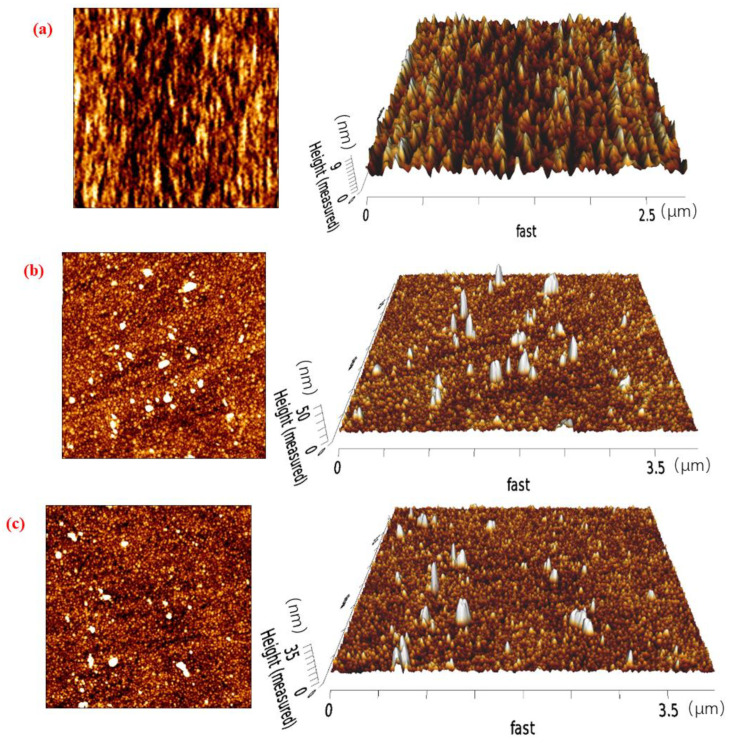
(**a**) Chip surface topography without adsorption of any reagent; (**b**,**c**) Chip surface topography of adsorbed mixed collectors with D-DA and D-BA.

As shown in Figure 7, (a) is the surface of the chip without adsorption of any reagent, (b) and (c) are respectively the surface of the chip on which a compound collector with different carboxyl group positions is adsorbed (the compound collector is D-DA, D-BA in turn), and each diagram includes the plane plan and 3D diagram of the chip surface. The atomic force microscopy (AFM) image of the compound collector with different carboxyl group positions after adsorption on the surface of the chip has obvious changes. The surface plan of the chip shows bright spots with different numbers, shapes, and sizes, and the average roughness of the locations where bright spots appear in the 3D image is greater than that of the original surface of the chip (9 nm). There are many mountain-shaped bright spots, indicating that the bright spots appearing on the surface of the chip are the compound collector adsorbed there. Comparing the AFM images of (b) and (c), it was found that more bright spots appeared on the surface of the chip treated with different compound collector solutions, and the adsorption amount could not be directly judged. However, compared with 3D images, it was found that the average height of bright spots adsorbed with D-DA was higher than that of D-BA, which also confirmed the special molecular topology of BA molecule, and echoed its conformation at the LRC/water interface.

### 2.3. Flotation Results

**Figure 8:**
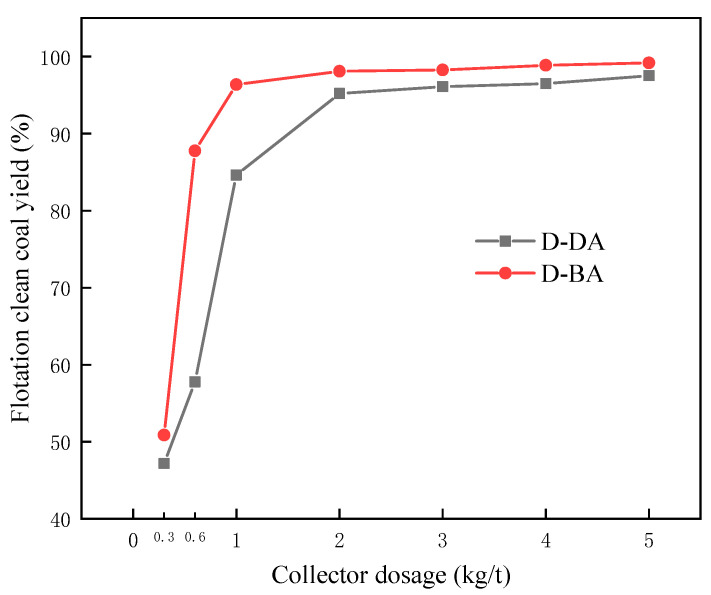
Flotation results of compound collectors with different carboxyl positions on pure coal samples.

Figure 8 shows the flotation results of pure coal samples with mixed collectors at different carboxyl group positions. As can be observed from the figure, as the quantity of the compound collector increases, the yield of flotation clean coal increases in a different amplitude, and at the same dosage, the yield of cleaned coal obtained by combining D-BA with carboxyl group at the carbon chain’s middle is higher than that obtained by combining D-DA with a carboxyl group at the carbon chain’s termination. Among them, when the dosage is 0.6 kg/t and 1 kg/t, there is a large gap between the two kinds of flotation clean coal yields, and the cleaned coal yields obtained by D-BA are 30% and 12% higher than those obtained by D-DA, respectively. Using a 2 kg/t dose, the yield of flotation refined coal has reached 98.11% (D-BA) and 95.21% (D-DA), and almost all useful minerals have come to the surface. In general, the D-BA with a carboxyl group at the carbon chain’s middle can increase the yield of cleaned coal than the D-DA with a carboxyl group at the carbon chain’s termination.

**Figure 9:**
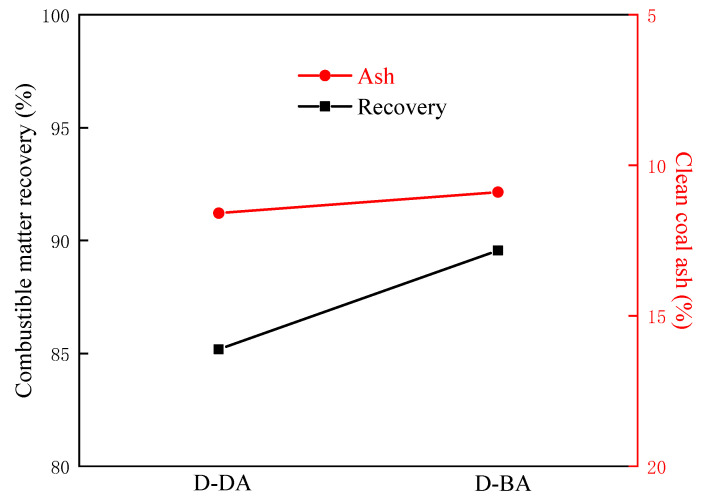
Flotation results of compound collectors with different carboxyl positions on actual coal samples.

In the pure coal flotation experiment, when the amount of collector reagent is more than 2 kg/t, a higher coal yield can be obtained, and with the increase in the amount of agent, the coal yield increases very slowly. Therefore, in the actual coal sample flotation test, the dose for the compound collector is set at 3 kg/t, and their flotation behavior on the actual LRC sample is investigated. Figure 9 displays the flotation results for actual coal samples using compound collectors with various carboxyl group positions. The chart shows that the combustible matter recovery occurs in the following order: D-BA (89.56%) > D-DA (85.18%), and the cleaned coal ash is in the order of D-DA (11.59%) > D-BA (10.89%). Under the same flotation conditions, the use of the D-BA can obtain a higher combustible matter recovery but can also obtain a lower cleaned coal ash content. To sum up, the order of the flotation effect of compound collectors with different carboxyl group positions on LRC is D-BA > D-DA. This also confirms that in the actual flotation process, the assembly behavior and final assembly structure of D-BA with the carboxyl group at the carbon chain’s middle are more beneficial to the improvement of flotation efficiency.

## 3. Materials and Experimental Methods

### 3.1. Materials

**Figure 10:**
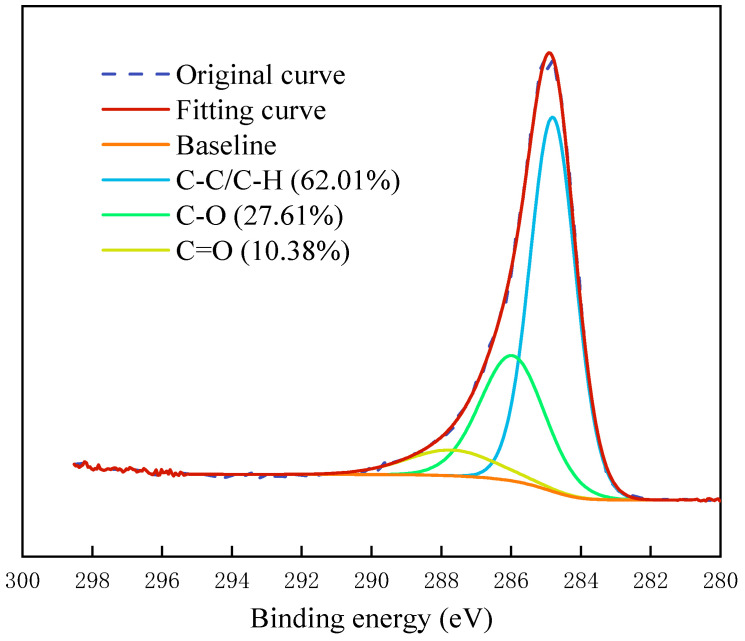
The C_1s_ fitting spectra of the pure coal sample.

In this investigation, two LRC samples, raw coal and block refined coal, were used for experiments. Ultra-low ash coal with an ash content of 2.38% was achieved as a pure coal sample through a float and dip experiment. Figure 10 shows the narrow sweep result of XPS C_1s_ of the pure coal sample. It could be seen that the hydrophilic functional group content was very high, which was a typical LRC, and the ratio of the oxygen-containing functional group content was about 3:1. The raw coal and pure coal samples were ground into powder and the −0.5 mm powder samples were obtained through the 0.5 mm sample screen for the flotation test. In other tests, since the equipment requires a smooth test surface, the corresponding coal chip was customized from the Biolin Company as a test object according to the XPS results of the coal samples, which was used to study the compound collectors’ assembly structure and behavior on the LRC surface.

This study mainly explored the influence mechanism of carboxylic acids with different carboxylic group positions on the compound collector at the LRC/water interface, so two different carboxylic acids were selected, one was decanoic acid (DA) with the carboxylic group at the carbon chain’s termination, and the other was 2-butyl octanoic acid (BA) with the carboxylic group at the carbon chain’s middle. The two carboxylic acids were combined with dodecane in a 1:4 mass ratio to obtain the compound collectors D-DA and D-BA.

### 3.2. Coarse-Grained Molecular Dynamics Simulation

#### 3.2.1. Models

**Figure 11:**
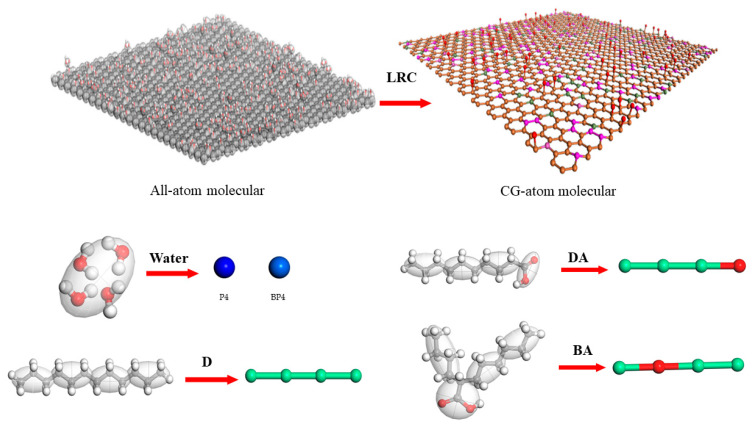
The mapping structures of different coarse-grained molecules (The red, gray, and white balls represent O, C, H respectively).

The LRC surface of 107 × 123 Å^2^ (X × Y) all-atomic molecules was constructed by randomly and proportionally grafting oxygen-containing functional groups on a single layer of graphene, using the modeling method described in the literature [5]. Then, graphene oxide/graphene is usually based on a Martini force field when studying mesoscopic dynamic behavior [28,29,30], which has a good matching and adaptability. Therefore, the Martini 2.0 force field [31,32] is also used in this investigation to simulate the CGMD of the LRC–compound collector–water system. The Martini model is based on the many-to-one mapping rule, which divides the coarse particles of the molecules in the system. According to the literature [19], the coarse-grained mapping of all-atomic LRC molecules, compound collector molecules, and water molecules was carried out and corresponding force fields were assigned. In addition, it should be noted that during the modeling process, BP4 coarse particles replaced P4 coarse particles with 10% [32] to prevent water from crystallizing at ambient temperature. Figure 11 shows the mapping structures of different coarse-grained molecules.

#### 3.2.2. Computational Method

For the CGMD computation, the Materials Studio 7.0 software’s Mesocite module was utilized. First, an initial LRC–water–compound collector coarse-grained system (X × Y × Z: 107 × 123 × 190 Å^3^) was used, where the system contained a 120 Å-thick vacuum plate to prevent periodic boundary conditions on the Z axis, and the mass ratio of dodecane molecules to carboxylic acid molecules in each compound collector was 4:1. The high energy of the initial system structure was avoided using the smart optimization strategy, and the CGMD with a 20 fs simulation time step and 1000 ns total time was calculated for the optimized structure. For specific simulation parameters and details such as the selection of ensemble and interaction calculation methods, see the Supplementary File S1.

### 3.3. Sum Frequency Generation Spectrometer

#### 3.3.1. SFG Experiments

Theoretical, technological, and analytical information on SFG has been widely disseminated elsewhere [33,34,35]. The sum-frequency spectrum system from EKSPLA (Lithuania) consisted of five main parts: picosecond laser system, frequency doubling system, optical parameter system, signal generation system, and acquisition system. The picosecond pulsed laser (wavelength 1064 nm) generated tunable infrared light (1000~4000 cm^−1^) and visible light (λ = 532 nm) for the SFG experiment after frequency doubling and three-parameter processes. The two beams of light were incident on the sample interface at the same time to produce sum-frequency signals. The incident light’s and the sum-frequency signal light’s polarization was regulated to be either the s or p direction (that is, the photoelectric field’s direction was either perpendicular or parallel to the incident plane), and the specific polarization of infrared light (s light or p light) to obtain the sum frequency vibration spectrum. The laser combination of these three specific polarization directions was referred to as a polarization combination of the frequency vibration spectrum. By convention, the names of the polarization combinations were given in the following order: sum frequency, visible, and infrared. For instance, ssp represented the sum-frequency signal light s polarization direction, visible incident laser s polarization direction, and infrared incident laser p polarization direction. Different polarization combinations give different spectral information. In this experiment, a reflective co-propagating configuration was used. The infrared and visible laser beams were incident at the sample interface at an incidence angle of (60° ± 1°) and (55° ± 1°), respectively. The delay was adjusted so that the two laser beams overlapped in space and time, that is, the sum-frequency signal light in the reflection direction was detected. The visible laser energy of the incident was about 200 μJ, and the infrared laser energy was about 150 μJ. The scanning step of infrared light was 2 cm^−1^, and each data point was obtained by the accumulation and average of 100 laser pulse measurement signals. The intensity of the SFG signal was normalized to the incident laser energy in the output spectrum (that is, SFG output energy values were obtained by dividing the infrared and visible incident laser energy and normalizing the gold film signal). The experiment was carried out in a super-clean working room with a constant temperature [(22.5 ± 0.5) °C] and constant humidity (40%).

The SFG test sample was a coal chip adsorbed by a mixed collector in a solution environment. The unadsorbed residual reagent solution on the surface of the chip was rinsed with ethanol and a large amount of ultra-pure water, then dried with high-purity nitrogen and placed on the sample table for the SFG test. The C–H vibration region (2800 cm^−1^ to 3000 cm^−1^) was mainly measured according to the composition of the compound collector. Two polarization states, ppp and ssp, were measured for each part of each sample. The fitting details of SFG spectra are shown in the Supplementary File S1.

#### 3.3.2. Calculation of Methyl Orientation Angle of the Interfacial Molecule

The biggest advantage of the SFG research interface was that it could not only correctly identify and vibrate spectral peaks by polarization analysis method, but it could also calculate the orientation angle of corresponding groups by the ratio of spectral peak intensity under different polarization combinations [36,37,38]. Generally, the angle between the molecular symmetry axis and the laboratory coordinate z-axis forward (that is, the interface normal) was defined as the molecular orientation angle θ, to quantitatively characterize the adsorption state of molecules on the interface, and it was an important parameter for studying the adsorption form of molecules on the interface. In this investigation, the orientation angles of methyl groups of different compound collectors on the coal chips’ surface were calculated.

First, the SFG signal strength is:

(2) $$I_{\left(\omega \right)}=\frac{8\pi^{3}\omega^{2}\sec^{2}\beta }{c^{3}n(\omega )n(\omega_{1})n(\omega_{2})}{\left|\chi_{eff}^{\left(2\right)}\right|}^{2}I_{1(\omega_{1})}I_{2(\omega_{2})}$$

where, ω, $\omega_{1}$, and $\omega_{2}$ are the frequencies of signal light, visible light, and infrared light respectively, β is the exit angle of signal light, $\chi_{eff}^{\left(2\right)}$ is the second-order effective polarizability of the interface, and c is the speed of light. $n(\omega_{i})$ is the wavelength-dependent refractive index of the bulk phase. It can be seen from Equation (1) that when the experimental configuration is unchanged, the SFG signal intensity directly reflects the second-order effective polarizability of the interface. To conveniently determine the orientation angle, the expression of $\chi_{eff}^{\left(2\right)}$ can often be expressed as a functional of the orientation angle [36], that is:

(3) $$\chi_{eff}^{\left(2\right)}=N_{s}d\left(\langle \cos\theta \rangle -c\langle \cos^{3}\theta \rangle \right)=N_{s}dr\left(\theta \right)$$

where $N_{s}$ is the interfacial molecular density, d is the strength factor, c is the generalized orientation parameter, and r(θ) is the orientation functional, which includes the information on the orientation angle of the molecular group and the orientation angle distribution. “〈〉” represents the ensemble average of the probability distribution function for the interface molecules in a certain orientation. The orientation angle is determined using the δ distribution function. When calculating the orientation angle, the c values and d values must be calculated first. For a given experimental system and experimental conditions, c and d are constant numbers. The methyl group of the $C_{3v}$ symmetry group under the polarization combination of ssp is utilized as a model to compute the c and d values. Thus

(4) $$\chi_{yyz}^{\left(2\right)}=\frac{1}{2}N_{s}\alpha_{ccc}^{\left(2\right)}\left[\left(1+r\right)\langle \cos\theta \rangle -\left(1-r\right)\langle \cos^{3}\theta \rangle \right]$$

where *r* is the methyl group’s depolarization ratio (here *r* = 3.4 [39]) and $\alpha_{ccc}^{\left(2\right)}$ is the non-zero hyperpolarizability tensor of the methyl symmetric stretching vibration mode. Through a series of conversions, the c and d values of the second-order polarizability of $C_{3v}$ symmetric methyl groups under the ssp polarization combination can be obtained in the following form:

(5) $$c=\frac{1-r}{1+r}$$

(6) $$d=L_{yy}(\omega )L_{yy}(\omega_{1})L_{zz}(\omega_{2})\sin\beta_{2}\alpha_{ccc}^{\left(2\right)}(1+r)$$

In the same way, the c and d values under the ppp polarization combination can be obtained.

After scanning the surface of the sample to obtain the SFG spectrum, the intensity values of each spectral peak of the SFG spectrum under different polarizations are obtained by fitting, and then the second-order nonlinear polarizability of a certain group under the combination of two polarizations is selected and its ratio (e.g., $\chi_{q,ppp}$/$\chi_{q,ssp}$) is calculated. Then the group’s orientation angle can be obtained by combining Formula (3). The general formula for calculating the orientation angle can be obtained from the above types:

(7) $$\frac{\chi_{q,ppp}}{\chi_{q,ssp}}=\frac{d_{ppp}\left(\langle \cos\theta \rangle -c_{ppp}\langle \cos^{3}\theta \rangle \right)}{d_{ssp}\left(\langle \cos\theta \rangle -c_{ssp}\langle \cos^{3}\theta \rangle \right)}$$

### 3.4. AFM Measurements

**Figure 12:**
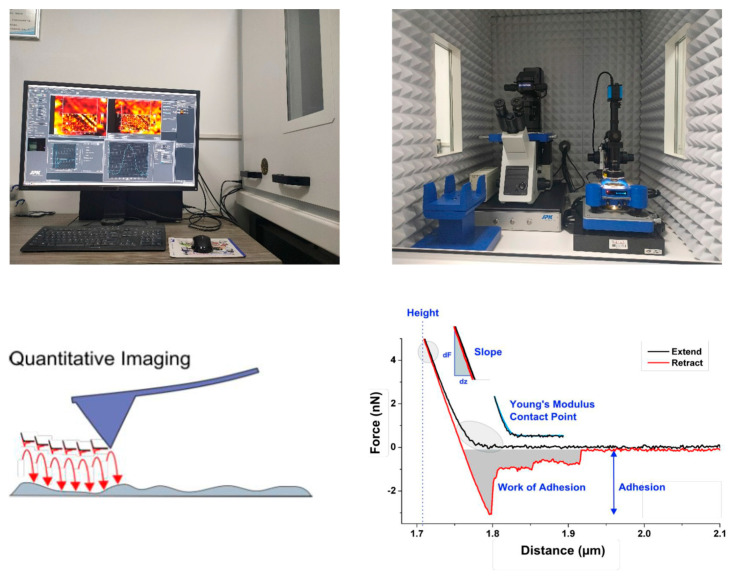
NanoWizard 4 AFM test device and scanning principle schematic diagram.

AFM technology can help to analyze the adsorption morphology of surfactants or polymers on mineral surfaces [22,40,41]. The samples were coal chips adsorbed by different kinds of compound collector solution, and the processing process was the same as that of the SFG test coal chips. NanoWizard Type 4 AFM (JPK, Berlin, Germany) was used to perform in situ imaging of the chip samples adsorbed with the collector in Quantitative Imaging mode (QI), and the chip surface without adsorbing any reagent was used as a blank control. QI mode is a mode based on the force spectrum, in which a complete force curve is recorded on every pixel of the sample. Because the probe is fully raised between each pixel, there is almost no lateral force in this mode. Figure 12 shows the NanoWizard 4 AFM test device and the schematic diagram of the scanning principle. The probe used was given a resonance frequency of 75 kHz, an elastic coefficient of 2.8 N/m, a Setpoint value of 0.2 V, and a scanning rate of 1.66 Hz.

### 3.5. Flotation Experiments

A 0.5 L XFD single-cell flotation machine was employed to carry out the LRC flotation test, and the flotation pulp concentration was 60 g/L. Firstly, the coal used for flotation was a pure coal sample, and the collection performance of the LRC with different saturation of hydrocarbon oil–fatty acid compound collector was mainly investigated. The addition amounts of each collector in the flotation process were 0.3, 0.6, 1, 2, 3, 4, and 5 kg/t, respectively, using sec-octanol as a frother; each addition was 0.3 kg/t. In the flotation process, the aeration was 0.25 m^3^/h, and the machine impeller speed was 1800 r/min. The pulp was mixed for three minutes, the compound collector was added and stirred for three minutes, and after adding the frother and stirring it for 30 s, opening the intake valve allowed the floating, cleaned coal to be collected for three minutes. The collected coal samples were filtered, dried, and weighed following flotation, and the yield of flotation-refined coal was computed.

Next, the pure coal sample was changed into the actual coal sample. In accordance with the pure coal sample’s flotational behavior, the appropriate compound collector addition system was selected. The dosage of other flotation reagents, pulp concentration, and parameters were the same as the flotation settings of the pure coal sample, and it was investigated how the effects of several types of compound collectors compared. At the end of flotation, to evaluate the ash content of coal samples, a Muffle furnace was used to dry, weigh, and burn the final collected floating cleaned coal and tailings. Ash content and the combustible matter recovery (Formula (8)) were calculated to assess the flotation effect:

(8) $$\mathrm{Combustible}\;\mathrm{matter}\;\mathrm{recovery}(\%)=\left(\frac{M_{C}\left(100-A_{C}\right)}{M_{F}\left(100-A_{F}\right)}\right)\times 100$$

In the formula, M represents the weight, A represents the ash content, and C and F respectively represent the flotation of cleaned coal and the feed.

## 4. Conclusions

In this work, the assembly behavior and structure of the compound collector with different carboxyl group positions at the LRC–water interface were investigated through CGMD combined with SFG. The CGMD results showed that the BA molecule interacts strongly with the D molecule as a whole, and the interaction distance is also closer. Therefore, when forming the final assembly structure, the first layer adsorption of the molecules in the D-BA was more parallel and ordered. The orientation distribution angle of carboxyl groups in BA molecules was larger, indicating that BA molecules with carboxyl groups in the middle of the carbon chain had a flatter spreading angle on the LRC surface as a whole and were more firmly adsorbed to the LRC surface in polar water phase than DA molecules with carboxyl groups at the carbon chain’s termination. SFG research found that the carboxyl group at the carbon chain’s termination had a greater impact on the displacement of the methyl/methylene symmetric stretching vibration peak, while the carboxyl group at the carbon chain’s middle had a more significant effect on the displacement of the methyl/methylene asymmetric stretching vibration peak. Moreover, after the D-BA treatment, the methyl group on the chip surface had a minor orientation angle, which was because the carboxyl group was in the carbon chain’s middle so the carbon chain on either side of the carboxyl group formed a certain angle. In general, D-BA spread more flatly on the LRC surface than D-DA, and the effective adsorption area of the assembled structure on the LRC surface was larger. AFM 3D scanning images showed that the average height of bright spots adsorbed with D-DA was higher than that of D-BA, which also confirmed the special molecular topology of the BA molecule and echoed its conformation at the LRC/water interface.

The flotation experiments of pure coal and actual coal samples indicated that the D-BA can obtain a higher combustible matter recovery and cleaned coal yield than the D-DA. This also confirmed that throughout the actual flotation procedure, the assembly behavior and final assembly structure of D-BA with the carboxyl group in the carbon chain’s middle at the LRC–water interface were more beneficial to the improvement of flotation efficiency, and it also expressed that the research on the interface assembly behavior and assembly structure can serve as a valuable resource and direction for the creation of effective compound collectors. The combination of SFG and CGMD is also an effective means to study interface assembly.

## Figures and Tables

**Table 1:** Assignments of sum-frequency vibration spectra of compound collectors with different carboxyl positions adsorbed on the surface of coal chip in different polarization.

Wavenumber	ppp Polarization State (cm^−1^)				ssp Polarization State (cm^−1^)			
Samples	CH_3, ss_	CH_3, as_	CH_2, ss_	CH_2, as_	CH_3, ss_	CH_3, as_	CH_2, ss_	CH_2, as_
Original	2875	2961	2852	2924	2869	2955	-	2924
D-DA	2867	2963	2829	2924	2860	2981	2840	2939
D-BA	2872	2962	2852	2923	2878	2954	2854	2930

## Data Availability

The original contributions presented in the study are included in the article/supplementary material, further inquiries can be directed to the corresponding author/s.

## Supplementary Materials

The following supporting information can be downloaded at: https://www.mdpi.com/article/10.3390/molecules29051034/s1.
