# Supplementary material for: Effect of Carboxyl Group Position on Assembly Behavior and Structure of Hydrocarbon Oil–Carboxylic Acid Compound Collector on Low-Rank Coal Surface: Sum-Frequency Vibration Spectroscopy and Coarse-Grained Molecular Dynamics Simulation Study
Source: Molecules. 2024 Feb 28;29(5):1034. doi: 10.3390/molecules29051034 (PMC10935132; doi:10.3390/molecules29051034)
Supplement: Supplementary file 1 [file molecules-29-01034-s001.zip › molecules-2834992-supplementary.pdf]

Supplementary

Simulation details of CGMD and Geometry Optimization:

First, parameters of the CG force field were fed into the Mesocite module, guided by the Martini 2.0 force field. Since the energy of the model in the initial structure was high, after the model was structured, each system used the Smart optimization algorithm (which came with the simulation software) to minimize the initial system energy, so as to avoid the interruption of dynamic simulation calculation due to excessive energy difference during calculation. Specifies the maximum number of geometric optimization cycles to be 1.0×10^5. If this number of cycles was reached, the calculation would stop even if the convergence criteria are not satisfied. CGMD calculation of 1000 ns was performed on the optimized structure, and the simulation time step was 20 fs. The department of NVT thermodynamic ensemble (steady count of particles N, unvarying volume V, and unchanging temperature T) and Nose thermostat (298 k) were chosen. Calculations for the Leonard-Jones non-bond and Coulomb interactions utilized Bead-based and Ewald summation techniques, respectively, initiating at 9 Å for Leonard-Jones non-bond interactions and 0 Å for Coulomb interactions, with a truncation distance of 12 Å. The Leonard-Jones non-bond interaction and Coulomb interaction were calculated using Bead based and Ewald summation methods, respectively, with starting points of 9 Å (non-bond interaction) and 0 Å (Coulomb interaction), and truncation distance of 12 Å. Specifies how often every 5×10^7 steps frame was written to the trace file. To reduce computational duration, the low-rank coal surface base model is established prior to dynamic calculation (except for randomly grafted carboxyl group and hydroxyl group).

Details of SFG spectrum fitting:

For the SFG spectrum fitting, from a mathematical point of view, the best fitting effect can be obtained by fitting the SFG spectrum of each polarization state separately. However, in the actual experiment, from a physical point of view, the intrinsic properties of the surface molecules such as vibration frequency and peak width of the same sample under the same experimental configuration do not change with the change of polarization state. Therefore, when fitting SFG spectra under different polarization states of the same sample, it is necessary to associate these fixed invariants, use global fitting in Origin software to associate their invariants, and then carry out spectral fitting. In the

spectral fitting, Lorentz model is used to fit, the function form is as follows:

$$L(x) = \frac{A}{\pi} \frac{\frac{1}{2}\Gamma}{(x - x_0)^2 + (\frac{1}{2}\Gamma)^2}$$

Where, x is the horizontal coordinate of the spectrum, x_0 is the position of the peak, A is the amplitude of the peak, and Γ is the half-width of the peak.
